# Can Diet Alter the Intestinal Barrier Permeability in Healthy People? A Systematic Review

**DOI:** 10.3390/nu16121871

**Published:** 2024-06-14

**Authors:** Daniele de Souza Marinho do Nascimento, Ana Carolina Costa Campos Mota, Maria Clara da Cruz Carvalho, Eva Débora de Oliveira Andrade, Érika Paula Silva Freitas de Oliveira, Liana Letícia Paulino Galvão, Bruna Leal Lima Maciel

**Affiliations:** 1 Post Graduate Program in Health Science, Center for Health Science, Federal University of Rio Grande do Norte, Natal 59078-970, Brazil; daniele.marinho@ufrn.br (D.d.S.M.d.N.); maria.carvalho.074@ufrn.edu.br (M.C.d.C.C.); evaandradenutrii@gmail.com (E.D.d.O.A.); liana.galvao.017@ufrn.edu.br (L.L.P.G.); 2 Post Graduate Program in Nutrition, Department of Nutrition, Federal University of Rio Grande do Norte, Natal 59078-970, Brazil; ana.costa.094@ufrn.edu.br (A.C.C.C.M.); erika.freitas@ufrn.br (É.P.S.F.d.O.); 3 Department of Nutrition, Center for Health Science, Federal University of Rio Grande do Norte, Natal 59078-970, Brazil

**Keywords:** diet, intestinal barrier permeability, healthy people

## Abstract

Dietary factors can modify the function of the intestinal barrier, causing permeability changes. This systematic review analyzed evidence on the link between diet or dietary interventions and changes in intestinal barrier permeability (IBP) in healthy individuals. A systematic search for primary studies was conducted using the virtual databases EMBASE, PubMed, Web of Science, CINAHL, and Scopus. This review adhered to PRISMA 2020 guidelines, assessing the methodological quality using the Newcastle–Ottawa scale for observational studies and ROB 2.0 for randomized clinical trials. Out of 3725 studies recovered, 12 were eligible for review. Chicory inulin and probiotics reduced IBP in adults with a moderate GRADE level of evidence. The opposite result was obtained with fructose, which increased IBP in adults, with a very low GRADE level of evidence. Only intervention studies with different dietary components were found, and few studies evaluated the effect of specific diets on the IBP. Thus, there was no strong evidence that diet or dietary interventions increase or decrease IBP in healthy individuals. Studies on this topic are necessary, with a low risk of bias and good quality of evidence generated, as there is still little knowledge on healthy populations.

## 1. Introduction

The intestinal epithelium’s barrier function is of significant protective function, as it prevents allergens, toxins, and infections from diffusing into the tissue and circulatory system [1,2]. An imbalance in the gastrointestinal microbiota and its activity can disrupt tight junctions, altering intestinal permeability and increasing the risk of illnesses [3]. The intestinal barrier is composed of the mucus layer, commensal bacteria, epithelial cells, and immune cells residing in the lamina propria [4].

The investigation of intestinal permeability, as an indicator of barrier function, can be performed using a variety of techniques, the most common of which is the measurement of probe molecule excretion in the urine. This method, for example, enables the examination of the size and concentration at which these molecules may cross the intestinal barrier, as seen in sugar tests with lactulose:mannitol. Another element is the measurement of circulating levels of mucosal injury markers, such as zonulin and LPS (lipopolysaccharide). Other approaches include in vitro measurements using cell lines or human biopsies, such as assessing the expression of several tight junction proteins, like claudins, occludins, and zonula occludens. Finally, endoscopic measures play a significant role in this situation [5].

Dietary components are known to significantly impact intestinal physiology, specifically modulating the integrity of the intestinal barrier. In this context, measures of intestinal permeability markers appear promising for studying the impact of diet on barrier function [6,7].

Increased intestinal permeability has been associated with the intake of Western diets, particularly when combined with illness and exposure to environmental variables [8]. These diets are rich in saturated fats, carbohydrates, and simple sugars, and low in fiber, especially insoluble fiber. Bacterial strains in the intestinal colon that feed on these fibers gradually disappear, while other strains alter their metabolism to degrade the mucus that protect the intestine [9,10], resulting in increased intestinal permeability.

The consumption of some specific food components and their metabolites can reinforce both the structure and function of the intestinal barrier [5], such as insoluble fiber and the phytochemicals quercetin and curcumin, for example, found in a Mediterranean or vegetarian diet [4]. The degradation of insoluble fibers by colonic strains produces short-chain fatty acids (SCFAs), essential for the nutrition of colonocytes [10]. These same microorganisms are responsible for initiating the differentiation of regulatory T cells (Tregs), and can also induce the production of mucus, activating the secretion of IL-22 by innate lymphoid cells (ILCs) and IgA [4,5], regulating the human immune system.

The composition of the intestinal microbiota constitutes the most unstable element of the intestinal barrier, playing a fundamental role in its homeostasis and functions. Several factors affect the composition of this microbiota, with complementary feeding in childhood, eating habits throughout life cycles, and lifestyle being determining factors [10].

Given the evidence supporting the importance of dietary components in maintaining the integrity of the intestinal barrier, it is critical to assess whether diet-related strategies can help maintain this barrier in healthy people. Therefore, this study aimed to analyze evidence on the link between diet or dietary interventions and changes in the intestinal barrier permeability (IBP) in healthy individuals.

## 2. Methods

### 2.1. Protocol Registration

On 16 February 2023, the review protocol was registered in the International Prospective Register of Systematic Reviews (PROSPERO) under the number CRD 42023400381, which may be accessed at https://www.crd.york.ac.uk/prospero/display_record.php?ID=CRD42023400381, accessed on 27 February 2023. Following registration, changes to the protocol were made and recorded to better tailor the work. This study was conducted following the 2020 Reporting for Systematic Reviews and Meta-Analyses (PRISMA) guidelines (Table S1) [11]. The guiding question was: Does diet alter the permeability of the intestinal barrier in healthy people? A systematic review.

### 2.2. Inclusion and Exclusion Criteria

The study’s inclusion and exclusion criteria were defined using the PI/ECO strategy: P (population)—healthy persons without age restrictions; I/E (intervention/exposure)—better diet reduces intestinal barrier permeability/dietary intervention with specific functional nutrients alters intestinal barrier permeability; C (comparator)—healthy population with poor-quality food and/or control group without any changes in intestinal barrier permeability; O (outcome)—improvement in permeability of the intestinal barrier, leading to improved health and nutritional status.

This review included observational and intervention studies published without date or language constraints in scientific journals that matched the eligibility criteria. Review studies, systematic reviews, case reports, books, conference proceedings, short communications, editorials, letters to the editor, theses, dissertations, studies without abstracts, preclinical studies, medicinal dietary supplements, pharmaceutical probiotics or studies with conflicts of interest and that were not associated with any specific food, and studies that evaluated intestinal permeability in athletes or people with any associated pathology/co-morbidity/overweight status were excluded.

### 2.3. Search Strategy

A complete virtual literature search was carried out in March 2023, utilizing the PubMed, Scopus, EMBASE, Web of Science, and CINAHL databases. Search strategies used terms indexed in Medical Subject Headings (MeSH) (Table S2), serving as a highly sensitive search strategy. Following a systematic search, the articles were entered into the RAYYAN software, accessed in https://www.rayyan.ai, on 1 March 2023 and any duplicates found were removed. Following the eligibility criteria, two separate authors (DSMN and ACCCM) conducted a first screening of the studies based on the information in their titles and abstracts, followed by an examination of the entire study. In the event of a conflict, a third reviewer was available for the final decision (BLLM).

### 2.4. Data Extraction

Data from the selected studies were extracted and summarized in an electronic spreadsheet, which contained the following information: authors, year of publication, title, type of study, study population (characterization), sample size, methodology applied, methods of evaluating intestinal permeability and food consumption, statistical analyses performed, results of observation or dietary intervention with intestinal permeability, conclusion, observation, and reference.

Data in studies were considered significant when they presented a significant mean difference with *p* < 0.05, that is, when the changes found in IBP, both in observational and intervention studies, had a significant difference in two periods or in two different groups.

### 2.5. Methodological Quality and Risk of Bias

Two independent reviewers (ACCCM and MCCC) assessed the methodological quality and risk of bias, which helped to determine the strength of the scientific evidence presented in the included studies. In cross-sectional studies, the adapted Newcastle–Ottawa scale was employed [12], and scores of 0–3, 4–6, or 7–9 were classed as “very high risk of bias”, “high risk”, or “high methodological quality”, respectively. Randomized clinical trial studies were reviewed using the Cochrane instrument (Cochrane risk of bias tool for randomized trials—RoB 2.0) [13], which evaluates fixed sets of prejudice domains, which can be judged as “low or high risk” or expressing “some concerns”.

### 2.6. Best Evidence Synthesis

The Grading of Recommendation, Assessment, Development, and Evaluation (GRADE) framework assessed the level of evidence for each outcome of interest in this study, classifying the quality of the evidence as high (it is unlikely that new studies will change the confidence in the effect estimate), moderate (new studies can impact our confidence), low (new studies can have an important impact on confidence, changing the effect estimate), or very low (when certainty is very limited, making any finding uncertain) [14].

## 3. Results

### 3.1. Search Selection

**Figure 1:**
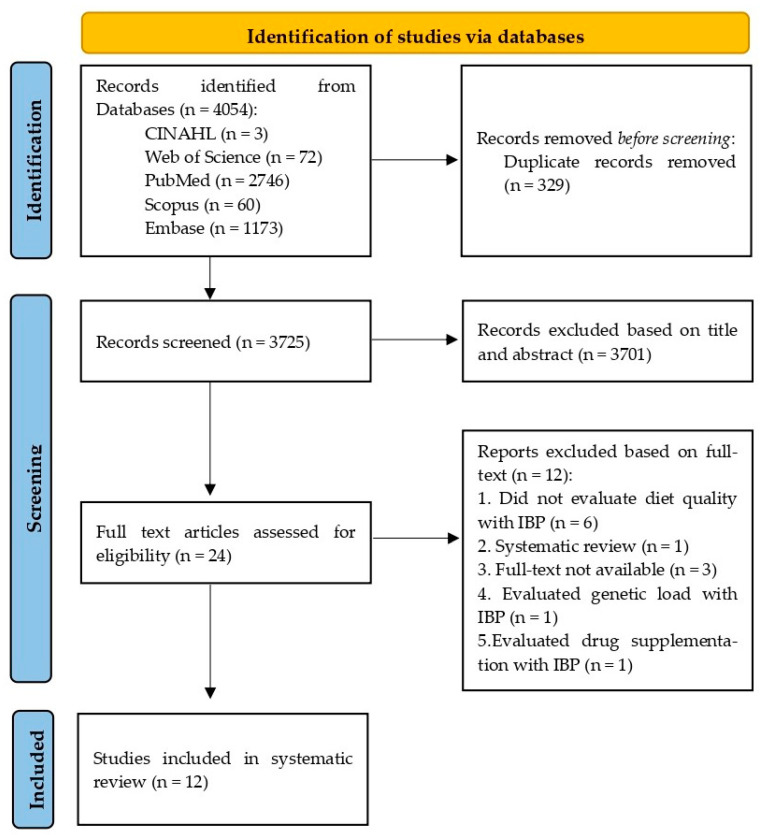
PRISMA flowchart of the included studies.

A virtual search in the databases, including all electronic search strategies, yielded 4054 records. After eliminating duplicates, 3725 publications’ titles and abstracts were evaluated, with 3701 eliminated because they did not match the eligibility criteria. After evaluating the studies in their entirety, 12 of the 24 publications selected for full text examination were removed: 6 were unrelated to the review theme; 3 lacked access to the complete text, despite attempts to contact the author; 1 investigated genetic load with intestinal permeability; 1 evaluated medication supplementation with intestinal permeability; and 1 was a review protocol. As a result, 12 papers qualified for the final assessment. Figure 1 shows the flowchart for the article screening process.

### 3.2. Studies and Population Characteristics

The characteristics of the included studies are shown in Table 1. The predominant study design was randomized double-blind (41.7%), all were published in English, and they were carried out primarily in Europe, followed by the United States. The sample size varied between 12 [15] and 100 [16] people, with the adult population (66.7%) being the most studied. Participants ranged from 1 to 8 months for infants, 6 to 15 years for children and adolescents, 18 to 57 years for adults, and 65 to 75 years for the elderly, with men being the most prevalent group represented.

### 3.3. Assessment of the Intestinal Barrier Permeability

The research retrieved various markers to determine the IBP. Table 2 displays the study results, the nutritional intervention used, and the primary findings on the relationship between food and IBP.

Three of the twelve studies included in the review focused on infants from a month and a half to nine months of age [17,18,19]. The lactulose:mannitol test was used as an IBP measure in one study [19], ECP in feces was analyzed in another [17], and absorption of human alpha-lactalbumin and bovine beta-lactoglobulin in full-term neonates was assessed in another [18]. Research that evaluated IBP with the introduction of complementary foods found that these foods did not cause changes when provided after 12 weeks of life [19], with no difference between breastfed or formula-fed infants (*p* > 0.05), or at 4 months [17] when a reduction in fecal PCE is observed during this period (1.0 ± 1.4 of 0.5 ± 0.9 Kg/mg of protein, *p* = 0.02), indicating a reduction in IBP. In respect to infants fed formula or breast milk, those breastfed presented a higher lactulose/mannitol ratio, indicating greater IBP. However, this difference resolved after 12 weeks of life, with no significant difference between them [19]. Kuitunen and collaborators [18] detected human alpha-lactalbumin from birth to the second month of life. In the second month, only 10% of the circulating total was found compared to the third and fourth days of life. Alpha-lactalbumin was not detected in the third month, and bovine beta-lactoglobulin was detected in the serum after the introduction of cow’s milk into the diet.

Two studies evaluated the consumption of probiotics associated with food, one using the strain of Lactobacillus acidophilus associated or not with honey in children and adolescents aged 6 to 15 years [22] and another using the strain of *Streptococcus thermophilus* ST10 associated with the consumption of gums in adults aged 21 to 57 [23]. The *Lactobacillus acidophilus* strain had the ability to restore the integrity of the intestinal barrier function, significantly reducing the urinary recovery of lactulose:mannitol when associated with honey (from 4.29 ± 0.63 to 2.19 ± 0.35, p = 0.01) or not associated with honey (from 4.13 ± 0.59 to 2.61 ± 0.28, *p* = 0.04) [22]. In turn, the *Streptococcus thermophilus* ST10 strain also reduced IBP in both the small intestine and large intestine, with a significant difference in the lactulose:mannitol ratio between the intervention and control groups after 30 days (from 0.021 to 0.014, *p* = 0.045—group intervention) and a significant difference in sucralose excretion after 30 days (from 35.8 mg to 27.9, *p* = 0.038—intervention group) [23].

Two studies investigated whether a high-fat diet, particularly saturated fats, altered the IBP in adults in the short term [20,21], and found no change in the proportion of recovery of urinary lactulose/mannitol and other markers, with no significant difference between the periods before and after intervention with the proposed diets (*p* > 0.05). Another study evaluated increased short-term fructose intake, which led to increased endotoxemia (*p* < 0.05), intestinal translocation of bacterial endotoxemia (*p* < 0.05) and altered IBP [15].

The other included studies investigated the effect prebiotics in the IBP (n = 4): fructooligosaccharides (FOS) [25], barley beta-glucans [26], chicory inulin-enriched pasta [24], and pectin derived from beetroot [16]. The study evaluating FOS measured urinary excretion of chromium EDTA (CrEDTA) to assess the permeability at the level of the large intestine, and there was no change in permeability (3.3 ± 0.2 in control group and 3.0 ± 0.3 in FOS group, *p* > 0.05). Two studies evaluated permeability at the small intestine level using the urinary recovery of lactulose/mannitol and found that barley beta-glucans [26] did not modify permeability (1.447 ± 1.606 and 1.497 ± 1.493, *p* = 0.949). In turn, inulin [24] decreased lactulose excretion, thereby reducing the lactulose/mannitol ratio, indicating a reduction in macromolecule absorption and the barrier permeability (lactulose/mannitol ratio 0.05; 0.04–0.09 in the control group and 0.03; 0.02–0.05 in the inulin group, *p* = 0.0012). Finally, Wilms et al. [16] observed that beetroot-derived pectin had no effect on permeability in any of the gastrointestinal segments studied (gastroduodenal; small, large and total intestines) or in the populations investigated (young adults and elderly) (*p* > 0.05).

### 3.4. Quality Assessment and Risk of Bias

The methodological quality of the included studies was assessed, and the full evaluation is provided in the Supplementary Material (Tables S3 and S4). Cross-sectional studies (n = 3) obtained scores ranging from 7 to 9, indicating high methodological quality. In turn, of the clinical trials (n = 9), two (n = 2) presented a low risk of bias, five (n = 5) presented some concerns, and two (n = 2) had a high risk of bias. 

### 3.5. Association between Diet and Intestinal Barrier Permeability (IBP)

Table 3 summarizes the relationship between exposures or dietary interventions and IBP, as well as the quality of the evidence generated. The GRADE framework classified the observational research as having low- and very low-quality evidence, but intervention studies were predominantly classified as having moderate-quality evidence.

Complementary feeding in infants (n = 3) was not associated with IBP, both in breastfed infants and in those who used formula, using the markers lactulose/mannitol, ECP, human alpha-lactalbumin, and bovine beta-lactoglobulin, generating a low quality of evidence [17,18,19].

Studies that evaluated the use of two different types of probiotics (n = 2) in different populations demonstrated a positive association in reducing the urinary recovery of lactulose/mannitol in adults, children (>6 y), and adolescents (<15 y), demonstrating a reduction in IBP, with a moderate quality of evidence [22,23].

Of the studies evaluating the increase in saturated fat intake (n = 2), in the short term, they did not demonstrate an association with the IBP, with no increase or decrease in urinary recovery of lactulose/mannitol, generating very low-quality evidence [20,21]. In the dietary intervention with fructose (n = 1), a negative association with IBP was observed, leading to its increase, with a very low quality of evidence [15].

Three studies [24,25,26] used prebiotics in dietary interventions in adults, while one used them in adults and the elderly [16]. The study that assessed the intervention with FOS [25] found no association with the IBP alteration and had moderate evidence quality. The study that used barley beta-glucans [26] showed no association with changes in the IBP, with a high quality of evidence. The study that worked with inulin [24] demonstrated a positive association in reducing the IBP after intervention, with a moderate quality of evidence. Finally, the intervention with beet pectin, in the study by Wilms et al. (2019) [16], showed no association with changes in IBP, with a moderate quality of evidence.

## 4. Discussion

In this systematic review, we evaluated whether diet or dietary interventions alter the function of the intestinal barrier by modifying its permeability in healthy persons. Furthermore, the methodologies and markers that evaluate dietary intervention in IBP were highlighted. Twelve research meeting the inclusion criteria were reviewed, including three observational studies and nine clinical trials.

Three studies evaluated intestinal permeability in infants and found high intestinal permeability in the first months of life [18,19]; the permeability decreased with age [17], but early introduction of complementary feeding was associated with greater intestinal permeability [19]. The infants’ digestive system is physiologically immature at birth, with greater permeability of the intestinal barrier [18], with the epithelium being permeable to human and foreign protein molecules in the first months of life. As the infant grows, its digestive system matures, strengthening the mucosa and decreasing the intestinal barrier permeability [17].

Two studies evaluated the application of a high-fat diet in the population and found no changes in intestinal permeability after a period of 5 [20] and 7 days [21] of intervention. Another study evaluated the intervention of a high-fructose diet [15], which showed compromised intestinal barrier function after a brief period of diet administration (three days of intervention, followed by a wash-out phase).

Three studies found decreased intestinal permeability associated with the use of food-associated probiotics [22,23] and prebiotics [24]. On the other hand, no significant changes in intestinal permeability were observed with the consumption of prebiotics in three studies [16,25,26]. Data related to supplementation with pre- and probiotics and improvement of intestinal permeability in the literature are inconsistent [27,28]. Supplementation with pre- and probiotics can be beneficial in terms of inflammation and intestinal microbiota [29] but can also contribute to increased IBP [25]. Fermentation with prebiotics increases the number of beneficial bacteria to intestinal health, but in the studies evaluated during this systematic review, only one demonstrated significant effects in reducing intestinal permeability [24]. 

The composition of the microbiota influences intestinal permeability as well as the host’s immune system. The microbiota’s beneficial symbiotic bacteria contribute to the barrier’s homeostasis by protecting against infections and boosting immune cell development [29]. When the bacterial composition changes due to changes in diet and lifestyle, there may be changes in human physiological functioning, leading to the development of pathologies and their aggravation [4]. Consuming a Western diet and unhealthy lifestyle habits results in dysbiosis, whereas consuming a Mediterranean or vegetarian diet and healthy lifestyle habits maintain homeostasis [30]. Dysbiosis, produced by changes in the microbiota composition, may result in increased mucosal permeation and intestinal and systemic inflammation, directly influencing the host’s health [31,32].

Thus, microbiota regulation is intimately related to diet quality. However, this relationship is difficult to assess, and knowledge of which specific foods or diets support the growth of a given bacteria is still evolving [30]. A fiber-rich diet, particularly insoluble fiber, supplies the bacteria in the colon with a fermentable component. Fermentation by beneficial symbiotic bacteria will provide energy to the colonocytes while producing gases and SCFAs. The SCFAs, in turn, decrease intraluminal pH, inhibiting pathogen colonization, in addition to reducing the solubilization of bile acids, allowing greater bioavailability of calcium and other components [4,33], supporting a thick layer of mucus in the intestinal mucosa [31,32], and producing anti-inflammatory interleukins [33]. 

Different methodologies to assess IBP in different segments were found in the studies retrieved in this review. Lactulose and mannitol were analyzed in the urine of individuals in eight studies [16,19,20,21,22,23,24,26]; of these, four studies included other saccharides in their methodologies [16,20,21,23]. Urine samples were collected for up to 5 h after ingestion of the lactulose and mannitol solution to assess gastric [16,20] and intestinal permeability [16,20,21,22,23,24], and up to 6 h to assess intestinal permeability [26]. Colon permeability was assessed for up to 24 h after ingestion of the sucralose solution [16,20,23]. A study of infants collected 2 mL of urine after 24 h of regular ingestion of a lactulose and mannitol solution [19].

The quality of the studies’ evidence was assessed as very low [20,21], low [19], moderate [16,22,23,24], and high [26]. Studies with very low and low quality of evidence presented a high risk of bias or some concerns for bias, and these studies did not present associations in their results.

Zonulin was evaluated in two studies [21,24], and no changes in the marker were found before and after the intervention period when using a high-fat diet [21]. However, a significant reduction in serum zonulin was found when prebiotics were used [24]. The studies obtained a very low [21] and moderate quality of evidence in the assessment [24].

Polyethylene glycol (PEG) absorption tests were performed in one study [21], with urine collected during 21 h after ingestion of the PEG solution, and no consistent change in intestinal permeability were detected. Bacterial endotoxin and serum lipopolysaccharide-binding protein (LBP) [15] were investigated in a single study with very low-quality evidence and high risk of bias.

Studies in infants have utilized eosinophilic cationic protein (ECP) [17], human alpha-lactalbumin (ALA), and serum bovine beta-lactoglobulin (BLG) [18], as well as the lactulose:mannitol ratio [19], all with a low quality of evidence, despite high methodological quality.

As observed in the present study, many gut permeability tests assess intestinal barrier permeability (IBP), including oral probes recovered in urine and fecal and plasma biomarkers. However, several considerations should be addressed when deciding which test to use, including cost, material availability, the population to be investigated, and available reading equipment. Multi-sugar tests are widely accepted and are mostly used to assess IBP throughout the intestine since they directly assess IBP. They are non-invasive, low-cost, and not metabolized but absorbed passively and eliminated in the urine [34]. Different probes evaluate specific gastrointestinal segments: sucrose evaluates gastroduodenal permeability; lactulose, L-rhamnose, and mannitol evaluate small intestine permeability; sucralose and erythritol monitor large intestine permeability. Urine PEG is used to evaluate small intestine motility and absorption, and CrEDTA can be used as a sensitive indicator of colonic intestinal permeability [35]. All these probes can be employed separately for each segment or jointly to assess paracellular or transcellular permeability and various gastrointestinal segments [36]. Although not directly reflecting intestinal permeation, plasma markers can also be used, such as zonulin, a physiological mediator of intestinal paracellular permeability regulation [37], and LBP, used to evaluate intestinal bacterial translocation [21]. Thus, although these tests seem redundant, they are complementary, especially concerning absorption oral probes, and chosen considering the study’s population and aims.

Our research and analysis revealed several articles that addressed changes in the intestinal microbiome or changes in IBP associated with co-morbidities. However, more needs to be known about the association between diet and IBP in healthy people. Furthermore, the review included a diverse range of research, using different food/nutrient interventions, different IBP markers, and different segments of the intestine to evaluate IBP, which made a meta-analysis impossible, limiting this review. However, strengths include rating the methodological quality of studies and the overall assessment of evidence of exposure with each IBP marker measurement.

## 5. Conclusions

There was no strong evidence that diet or dietary interventions increase or decrease intestinal barrier permeability in healthy individuals. The observational studies found were related to healthy infants together with the introduction of complementary foods. These showed that complementary foods did not influence permeability if inserted from 3 months of age, and that before that, IBP is more related to physiological maturation than dietary interference. In other populations, only intervention studies with different dietary components were found, but few studies evaluated the effect of different diets on the IBP. Probiotics given in food and inulin were positively associated with IBP reduction. Studies on this topic are necessary, with a low risk of bias and a good quality of evidence generated, as there is still little research on healthy populations.

## Figures and Tables

**Table 1:** Characteristics of the studies included in this systematic review (n = 12).

Study Design	Authors (Year)	Country	Sample Size	Population
Descriptive cross-sectional	Amarri et al. (2006) [17]	Italy	22	Infants
	Kuitunen et al. (1994) [18]	Finland	20	Infants
	Weaver (1988) [19]	England and Gambia	77	Infants
Prospective intervention study	Bowser et al. (2020) [20]	USA	13	Men
	Nier et al., (2019) [15]	Áustria	12	Men and women
	Ott et al. (2018) [21]	Germany	25	Men
Randomized	Mohammad et al. (2007) [22]	Egypt	24	Boys and girls
Randomized double-blind	Del Piano et al. (2014) [23]	Italy	25	Men and women
Randomized double-blind crossover	Russo et al. (2012) [24]	Italy	20	Men
	Ten Bruggencate et al. (2005) [25]	The Netherlands	34	Men
Randomized double-blind—placebo-controlled	Skouroliakou et al. (2016) [26]	Greece	23	Men
	Wilms et al. (2019) [16]	The Netherlands	100	Men and women

**Table 2:** Characteristics of the included studies (n = 12), considering the method for intestinal permeability assessment, intervention characteristics, duration of the intervention, and the main results.

Reference of Study	Intestinal Permeability Method of Assessment	Intervention Characteristics (Treatment and Control)	Duration of the Intervention or Observation	Main Results
Amarri et al., (2006) [17]	Eosinophil cationic protein (ECP), feces	-	5 months of observation	The ECP reduced significantly during the first months of weaning (*p* = 0.02), although there was an inclination for these parameters to increase at the end of the 9-month period
Bowser et al., (2020) [20]	Four sugar probes: sucralose, mannitol, sucrose, and lactulose, urine	The volunteers were fed a control diet (55% carbohydrates, 30% fats, and 15% proteins) for two weeks; thereafter, they were fed an isocaloric, high fat diet for 5 days (30% carbohydrates, 55% fats and 15% proteins)	21 days	The high-fat diet (HFD) had no effect on intestinal permeability (paired *t*-test, *p* = 0.05), while fasting endotoxin levels rose twofold (*p* = 0.04)
Del Piano et al., (2014) [23]	Lactulose/mannitol test and sucralose concentration, urine	All volunteers were provided with 30 sachets containing either the gelling complex (250 mg of tara gum, 1 billion viable cells of *S. thermophilus* ST10 and 2.20 g of maltodextrin) or a placebo (2.50 g of maltodextrin)	30 days	After 30 days, the L/M ratio was significantly reduced in the active group compared with the placebo group (from 0.021 to 0.014, *t*-test, *p* = 0.045). The sucralose concentration decreased from 35.8 mg at baseline to 27.9 mg and 29.1 mg after 30 and 45 days, respectively (*p* = 0.038 and *p* = 0.026 compared with the placebo)
Kuitunen et al., (1994) [18]	Human alfa-lactalbumin (ALA) and bovine beta-lactoglobulin (BLG), serum	-	Average 8 months of observation	During the first few months after birth, the intestinal epithelium of infants is permeable to human and foreign protein fragments (Mann– Whitney U test)
Mohammad et al., (2007) [22]	Lactulose/mannitol test, urine	Volunteers were separated into four groups: (1) *Lactobacillus acidophilus* (L1a); (2) honey alone; (3) L1a combined with honey; (4) Control—porridge made from wheat flour and sugar cane	42 days	When compared to the mean baseline value, the supplement significantly decreased urinary recovery of lactulose:mannitol when combined with honey (from 4.29 ± 0.63 to 2.19 ± 0.35, *p* = 0.01) or not combined with honey (from 4.13 ± 0.59 to 2.61 ± 0.28, *p* = 0.04) (ANOVA, *p* < 0.05)
Nier et al., (2019) [15]	Endotoxin and lipopolysaccharide binding protein (LBP), serum	All volunteers received a standardized diet which complex carbohydrates for four days; thereafter, they received a fructose or glucose diet for three days (25% of total calorie intake)	7 days	When volunteers consumed the fructose-enriched diet for three days, plasma endotoxin concentrations increased significantly compared to their standardized diet (Wilcoxon test and Friedman test)
Ott et al., (2018) [21]	Sugar and polyethylene glycol (PEG) absorption tests (performed in parallel); measurement of zonulin in plasma	All volunteers were subjected to an overfeeding program (the inclusion of dairy cream generated a surplus of 1000 Kcal/day)	7 days	The high-fat diet had no impact on intestinal permeability in any permeability assessment
Russo et al., (2012) [24]	Lactulose/mannitol test, urine Zonulin, serum, and feces	All subjects underwent a baseline evaluation followed by two 5-week trial periods: inulin-enriched pasta or a control pasta diet (100 g/d = 11.0 and 1.4 g/d of fructans, respectively).	5-week study periods followed by an 8-week washout period in between and a 2-week run-in phase.	The L/M ratio was significantly distinct between the baseline (0.05; 0.02–0.10), control pasta (0.05; 0.04–0.09) and inulin-enriched pasta (0.03; 0.02–0.05) diets (Friedman test, *p* = 0.0012). A significant difference was identified between the inulin-enriched pasta group compared to the baseline and control pasta groups (Dunn’s post hoc test, *p* < 0.05)
Skouroliakou et al., (2016) [26]	Lactulose/mannitol test, urine	Subjects: flour fortified with barley balance Placebo: flour not fortified with barley balance	30 days	There was no difference in the L/M ratio between the groups studied. Beginning of the intervention (95% IC; −0.23, 0.81); the end of the intervention (95% IC; −1.69, 1.59) (linear regression)
Ten Bruggencate et al., (2005) [25]	Chromium EDTA (CrEDTA) excretion, urine	Subjects: ingested lemonade with 20 g of fructooligosaccharides (FOS) Placebo: ingested lemonade with 6 g/day of sucrose This dose was divided into three daily lemonade servings	Two 2-week supplement periods were used, with a 2-week washout period in between.	Student’s *t*-test. Urinary CrEDTA excretion did not differ between the 2 periods (*t*-test)
Weaver, (1988) [19]	Lactulose/mannitol test, urine	-	Follow-up at 6, 12 and 18 weeks of life	At 6 weeks of age, infants fed cows’ milk formula exhibited greater urinary lactulose:mannitol excretion ratios compared to breast-fed infants (Mann–Whitney U-test, *p* < 0.05). Cow’s milk formulas feeding was linked to higher intestinal permeability than breast feeding in 6-week-old infants
Wilms et al., (2019) [16]	Five sugar probes: lactulose, mannitol, sucrose, sucralose and erythritol, urine Ussing chamber, tissue samples from the sigmoid colon	Subjects: 15 g/day of sugar beet-derived pectin Placebo: 15 g/day of maltodextrin Both products were supplemented as dry powders, free of unpleasant tastes and odors, in single-dose 7.5 g sachets	Twice daily for 4 weeks	There was no significant difference in urinary sucrose excretion or lactulose:mannitol ratio, both from 0 to 5 h, between 4 weeks of pectin supplementation and placebo in young or elderly adults (linear mixed models and correction for baseline, *p* ≥ 0.861). The 5–24 h urinary sucralose/erythritol ratio and 0–24 h urinary sucralose/erythritol ratio were not significantly different between four weeks of pectin vs. placebo supplementation in both young adults and the elderly (linear mixed models and correction for baseline values, *p* ≥ 0.130). TEER in unstressed and stressed biopsies did not significantly change between four weeks of pectin versus placebo supplementation in elderly or young adults (linear nixed models and correction for baseline values, *p* ≥ 0.226). There was no significant difference in luminal fluorescein concentrations in unstressed and stressed biopsies after four weeks of pectin vs. placebo supplementation in both young adults and the elderly (linear mixed models and correction for baseline values, *p* ≥ 0.164).

**Table 3:** Summary of the evidence on associations between dietary intervention and intestinal barrier permeability (IBP) assessed for each IBP marker, considering the quality of the evidence assessed by the GRADE framework.

Exposure or Intervention	Outcome	Number of Studied Groups (Total Participants)	Quality of Evidence	Evidence Summary
Complementary feeding in breast-fed versus formula-fed infants	Alteration of IBP—lactulose/mannitol, ECP, *α*-lactalbumin, and bovine β-lactoglobulin	3 (22 + 77 + 20 = 119 infants)	⊕⊕•• Low due to inconsistency of results, low sample size, and non-representativeness	No association
Use of probiotics	Alteration of IBP— lactulose/mannitol and sucralose	2 (24 teenagers/childreen and 25 adults)	⊕⊕⊕• Moderate due to missing outcome data	Positive association
Short-term diet high in saturated fats	Alteration of IBP— lactulose/mannitol	2 (38 adults)	⊕••• Very low due to bias in randomization process, deviations from intended interventions, and selection of the reported result	No association
Increased short-term fructose intake	Alteration of IBP— lactulose/mannitol	1 (12 adults)	⊕••• Very low due to bias in randomization process, deviations from intended interventions, and measurement of the outcome	Negative association
Prebiotic intake: fructooligosaccharides (FOS)	Alteration of IBP— excretion of Cr-EDTA	1 (34 adults)	⊕⊕⊕• Moderate due to bias in selection of the reported result	No association
Prebiotic intake: barley beta-glucans	Alteration of IBP— lactulose/mannitol	1 (23 adults)	⊕⊕⊕⊕ High-grade	No association
Prebiotic intake: chicory inulin	Alteration of IBP—lactulose/mannitol	1 (20 adults)	⊕⊕⊕• Moderate due to bias in randomization process and selection of the reported result	Positive association
Prebiotic intake: beet pectin	Alteration of IBP— multi-sugar test	1 (52 adults and 48 elderly)	⊕⊕⊕• Moderate due to bias in selection of the reported result	No association

⊕•••: very low grade; ⊕⊕••: low grade; ⊕⊕⊕•: moderate grade; ⊕⊕⊕⊕: high grade.

## Data Availability

All the data from this study are available in the manuscript and in Supplementary Materials.

## Supplementary Materials

The following supporting information can be downloaded at: https://www.mdpi.com/article/10.3390/nu16121871/s1, Table S1: PRISMA checklist; Table S2: Full electronic search strategy for EMBASE, PubMed, Web of Science, CINAHL and Scopus databases. Table S3: Assessment of the methodological quality of cross-sectional studies using the modified Newcastle–Ottawa scale; Table S4: Assessment of the methodological quality of randomized controlled trials using the Cochrane risk of bias tool (RoB 2.0).
