# Supplementary material for: Can Diet Alter the Intestinal Barrier Permeability in Healthy People? A Systematic Review
Source: Nutrients. 2024 Jun 14;16(12):1871. doi: 10.3390/nu16121871 (PMC11206284; doi:10.3390/nu16121871)
Supplement: Supplementary file 1 [file nutrients-16-01871-s001.zip › nutrients-3018041-supplementary.pdf]

Supplementary Materials

Table S1. PRISMA checklist

Section and Topic	Item #	Checklist item	Location where item is reported
**TITLE**			
Title	1	Identify the report as a systematic review.	Page 1
**ABSTRACT**			
Abstract	2	See the PRISMA 2020 for Abstracts checklist.	Ok
**INTRODUCTION**			
Rationale	3	Describe the rationale for the review in the context of existing knowledge.	Pages 1,2
Objectives	4	Provide an explicit statement of the objective(s) or question(s) the review addresses.	Page 2
**METHODS**			
Eligibility criteria	5	Specify the inclusion and exclusion criteria for the review and how studies were grouped for the syntheses.	Page 3
Information sources	6	Specify all databases, registers, websites, organisations, reference lists and other sources searched or consulted to identify studies. Specify the date when each source was last searched or consulted.	Page 3
Search strategy	7	Present the full search strategies for all databases, registers and websites, including any filters and limits used.	Supplemen - tary material
Selection process	8	Specify the methods used to decide whether a study met the inclusion criteria of the review, including how many reviewers screened each record and each report retrieved, whether they worked independently, and if applicable, details of automation tools used in the process.	Page 3
Data collection process	9	Specify the methods used to collect data from reports, including how many reviewers collected data from each report, whether they worked independently, any processes for obtaining or confirming data from study investigators, and if applicable, details of automation tools used in the process.	Page 3
Data items	10a	List and define all outcomes for which data were sought. Specify whether all results that were compatible with each outcome domain in each study were sought (e.g. for all measures, time points, analyses), and if not, the methods used to decide which results to collect.	Page 3
	10b	List and define all other variables for which data were sought (e.g. participant and intervention characteristics, funding sources). Describe any assumptions made about any missing or unclear information.	Page 3
Study risk of bias assessment	11	Specify the methods used to assess risk of bias in the included studies, including details of the tool(s) used, how many reviewers assessed each study and whether they worked independently, and if applicable, details of automation tools used in the process.	Page 3
Effect measures	12	Specify for each outcome the effect measure(s) (e.g. risk ratio, mean difference) used in the synthesis or presentation of results.	Page 3
Synthesis methods	13a	Describe the processes used to decide which studies were eligible for each synthesis (e.g. tabulating the study intervention characteristics and comparing against the planned groups for each synthesis (item #5)).	Page 3
	13b	Describe any methods required to prepare the data for presentation or synthesis, such as handling of missing summary statistics, or data conversions.	Page 3
	13c	Describe any methods used to tabulate or visually display results of individual studies and syntheses.	Page 3
	13d	Describe any methods used to synthesize results and provide a rationale for the choice(s). If meta-analysis was performed, describe the model(s), method(s) to identify the presence and extent of statistical heterogeneity, and software package(s) used.	Page 3
	13e	Describe any methods used to explore possible causes of heterogeneity among study results (e.g. subgroup analysis, meta-regression).	Not applicable
	13f	Describe any sensitivity analyses conducted to assess robustness of the synthesized results.	Page 4
Reporting bias assessment	14	Describe any methods used to assess risk of bias due to missing results in a synthesis (arising from reporting biases).	Page 3
Certainty assessment	15	Describe any methods used to assess certainty (or confidence) in the body of evidence for an outcome.	Page 4
**RESULTS**			
Study selection	16a	Describe the results of the search and selection process, from the number of records identified in the search to the number of studies included in the review, ideally using a flow diagram.	Pages 4
	16b	Cite studies that might appear to meet the inclusion criteria, but which were excluded, and explain why they were excluded.	Page 4
Study characteristics	17	Cite each included study and present its characteristics.	Page 5
Risk of bias in studies	18	Present assessments of risk of bias for each included study.	Page 9 and Supplemen- tary material
Results of individual studies	19	For all outcomes, present, for each study: (a) summary statistics for each group (where appropriate) and (b) an effect estimate and its precision (e.g. confidence/credible interval), ideally using structured tables or plots.	Pages 6-9
Results of syntheses	20a	For each synthesis, briefly summarise the characteristics and risk of bias among contributing studies.	Pages 6-10
	20b	Present results of all statistical syntheses conducted. If meta-analysis was done, present for each the summary estimate and its precision (e.g. confidence/credible interval) and measures of statistical heterogeneity. If comparing groups, describe the direction of the effect.	Pages 6-10
	20c	Present results of all investigations of possible causes of heterogeneity among study results.	Not applicable
	20d	Present results of all sensitivity analyses conducted to assess the robustness of the synthesized results.	Pages 9,10
Reporting biases	21	Present assessments of risk of bias due to missing results (arising from reporting biases) for each synthesis assessed.	Page 9 and Supplemen- tary material
Certainty of evidence	22	Present assessments of certainty (or confidence) in the body of evidence for each outcome assessed.	Pages 10,11
**DISCUSSION**			
Discussion	23a	Provide a general interpretation of the results in the context of other evidence.	Pages 11,12
	23b	Discuss any limitations of the evidence included in the review.	Page 11,12
	23c	Discuss any limitations of the review processes used.	Page 12
	23d	Discuss implications of the results for practice, policy, and future research.	Pages 11,12
**OTHER INFORMATION**			
Registration and protocol	24a	Provide registration information for the review, including register name and registration number, or state that the review was not registered.	Page 2
	24b	Indicate where the review protocol can be accessed, or state that a protocol was not prepared.	Page 2
	24c	Describe and explain any amendments to information provided at registration or in the protocol.	Page 2
Support	25	Describe sources of financial or non-financial support for the review, and the role of the funders or sponsors in the review.	Page 12
Competing interests	26	Declare any competing interests of review authors.	Page 12
Availability of data, code and other materials	27	Report which of the following are publicly available and where they can be found: template data collection forms; data extracted from included studies; data used for all analyses; analytic code; any other materials used in the review.	Supplemen- tary material

From: Page MJ, McKenzie JE, Bossuyt PM, Boutron I, Hoffmann TC, Mulrow CD, et al. The PRISMA 2020 statement: an updated guideline for reporting systematic reviews. BMJ 2021;372:n71. doi: 10.1136/bmj.n71

Table S2. Full electronic search strategy for EMBASE, PubMed, Web of Science, CINAHL and Scopus databases.

**Databases**	**Search strategy**
EMBASE, PubMed, Web of Science and CINAHL	(intestinal barrier permeability OR intestinal permeability OR intestinal barrier OR Permeability OR Permeabilities OR Cell Membrane Permeability OR Permeability, Cell Membrane OR intestinal permeability OR gut barrier OR intestines) AND (diet quality OR dietary pattern OR diet index OR diet quality OR dietary assessment OR dietary indicators OR diet OR diets OR nutritional status OR Status, Nutritional OR Nutrition Status OR Status, Nutrition) AND (healthy people)
Scopus	(“intestinal barrier permeability” OR “intestinal permeability” OR “intestinal barrier” OR Permeability OR Permeabilities OR “Cell Membrane Permeability” OR “Permeability, Cell Membrane” OR “intestinal permeability” OR “gut barrier” OR intestines) AND (“diet quality” OR “dietary pattern” OR “diet index” OR “diet quality” OR “dietary assessment” OR “dietary indicators” OR diet OR diets OR “nutritional status” OR “Status, Nutritional” OR “Nutrition Status” OR “Status, Nutrition”) AND (“healthy people”)

Table S3. Assessment of the methodological quality of cross-sectional studies using the Modified Newcastle Ottawa Scale.

Modified Newcastle Ottawa Quality Assessment Scale								
	**Selection**				**Comparability**	**Outcome**		
**Study ID**	Representativeness of the sample (Maximum: ★)	Sample size (Maximum:★)	Non-respondents (Maximum:★)	Ascertainment of exposure (Maximum:★★)	The subjects in different outcome groups are comparable, based on the study design or analysis. Confounding factors are controlled (Maximum: ★★)	Assessment of outcome (Maximum:★★)	Statistical test (Maximum:★)	**Total score (out of 10)**
Kuitunen et al., 1994	-	-	-	★★	★★	★★	★	******* (7)
Weaver, 1988	★	-	-	★★	★	★★	★	******* (7)
Amarri et al., 2006	★	-	★	★★	★★	★★	★	********* (9)

Risk of bias (RoB) measured using the Newcastle Ottawa Quality Assessment Scale (adapted for cross sectional studies), averaged per item. In general, more stars denote higher quality. A study can be awarded a maximum of one star for each item within the ‘Selection’ and ‘Outcome’ categories. a A maximum of four stars can be given for ‘Selection’. ‘Representativeness’ is awarded a star if the cohort is truly or somewhat representative of the population of interest. For selection of the non-exposed cohort, a star is awarded if it is drawn from the same population as the exposed cohort. Exposure is satisfactorily ascertained if data are collected from a secure record. b A maximum of two stars can be given for ‘Comparability’, one each for controlling of two important confounders in either the design or analysis phase. c A maximum of three stars can be given for ‘Outcome’. ‘Assessment of outcome’ is awarded a star if the outcomes were assessed by independent blind assessment or record linkage. The duration of follow-up was considered adequate if it was long enough for the outcomes to occur. Completeness of follow-up was considered adequate if all patients were accounted for or if the number lost to follow-up was sufficiently low to be unlikely to introduce bias.

Caption:
7-9: has high quality
4-6: high risk of bias
0-3: very high risk of bias.

Table S4. Assessment of the methodological quality of randomized controlled trials using the Cochrane risk of bias tool (RoB 2.0).

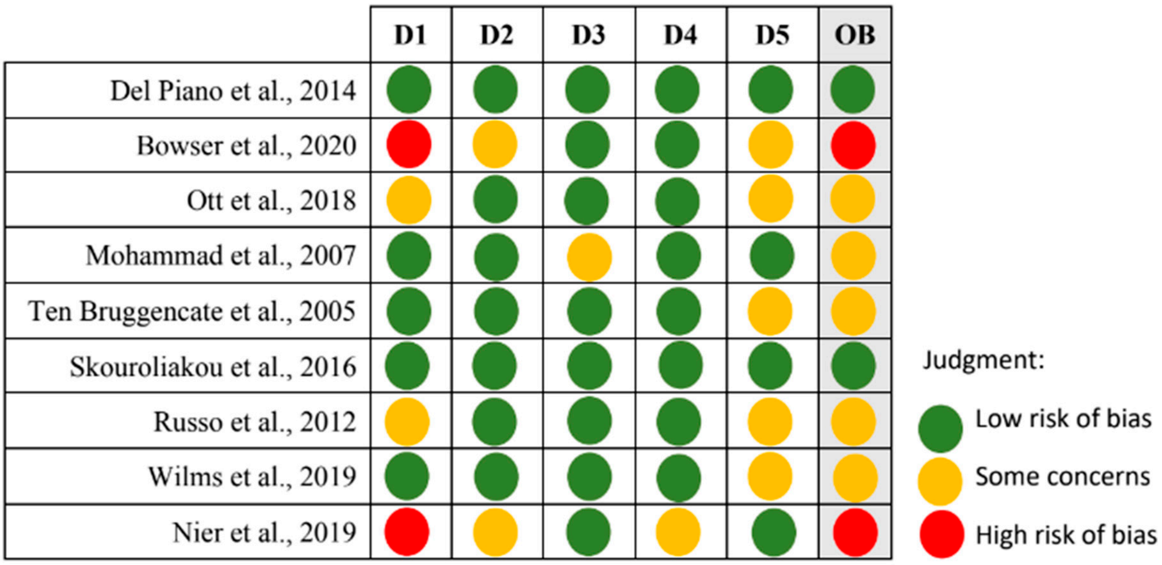

Domains:
D1: Bias arising from the randomisation process
D2: Bias due to deviations from intended interventions
D3: Bias due to missing outcome data
D4: Bias in measurement of the outcome
D5: Bias in selection of the reported result
OB: Overall bias

Risk of bias (RoB) measured using Cochrane risk-of-bias tool for randomized trials (RoB 2.0)
